# Old dogs, new trick: classic cancer therapies activate cGAS

**DOI:** 10.1038/s41422-020-0346-1

**Published:** 2020-06-15

**Authors:** Seoyun Yum, Minghao Li, Zhijian J. Chen

**Affiliations:** 1 Department of Molecular Biology and Center for Inflammation Research, University of Texas Southwestern Medical Center, Dallas, TX 75390 USA; 2 Howard Hughes Medical Institute, Chevy Chase, MD 20815 USA

**Keywords:** Innate immunity, Cancer microenvironment

## Abstract

The discovery of cancer immune surveillance and immunotherapy has opened up a new era of cancer treatment. Immunotherapies modulate a patient’s immune system to specifically eliminate cancer cells; thus, it is considered a very different approach from classic cancer therapies that usually induce DNA damage to cause cell death in a cell-intrinsic manner. However, recent studies have revealed that classic cancer therapies such as radiotherapy and chemotherapy also elicit antitumor immunity, which plays an essential role in their therapeutic efficacy. The cytosolic DNA sensor cyclic GMP-AMP synthase (cGAS) and the downstream effector Stimulator of Interferon Genes (STING) have been determined to be critical for this interplay. Here, we review the antitumor roles of the cGAS-STING pathway during tumorigenesis, cancer immune surveillance, and cancer therapies. We also highlight classic cancer therapies that elicit antitumor immune responses through cGAS activation.

## Introduction

Humanity’s battle against cancer has been ongoing for thousands of years with surgical removal of tumors being the first treatment recorded in Ancient Egypt.^1^ While surgery is still a first line treatment for cancer in modern times, it does not prevent systemic tumors and is limited by tumor accessibility and location. Beginning in the 20th century, classic cancer therapies that cause robust DNA damage and cell death became available. Classic therapies such as radiotherapy and chemotherapy became the major cancer treatments performed in the clinic; nevertheless, not all cancers respond to classic therapies, driving research towards new therapeutic strategies.

More recently, rapid progress has been made in the field of cancer immunology. The theory of cancer immune surveillance was formed in the late 20th century, suggesting that the immune system can identify and kill cancer cells.^2^ This idea was later confirmed upon detection of tumor antigen-specific CD8^+^ T cells in patients and led to the development of cancer immunotherapies such as immune checkpoint blockade.^3–5^ How can the immune system be activated by cancer cells in the absence of an infection? Cyclic GMP-AMP synthase (cGAS) is a cytosolic DNA sensor and was originally found to sense pathogen DNA during infection. Subsequent studies revealed that cGAS also detects tumor-derived DNA, initiating antitumor immunity. Moreover, cGAS provides additional antitumor roles by detecting DNA damage in premalignant cells or in cancer cells treated with classic cancer therapies. In this review, we provide an overview of the antitumor mechanisms of cGAS-mediated immune responses.

## The cGAS-STING pathway

**Fig. 1:**
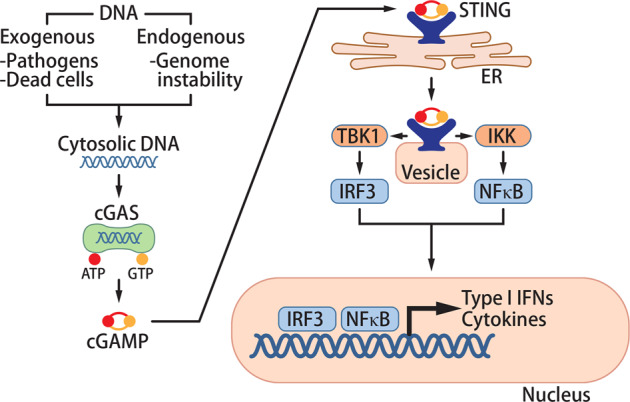
The cGAS-STING pathway. Abnormal localization of DNA in the cytosol elicits an immune response through the cGAS-STING pathway. Cytosolic DNA derives from exogenous (pathogens and dead cells) and endogenous (genome instability or mitochondrial damage) sources. cGAS binds to DNA in the cytosol and converts ATP and GTP into 2′3′-cGAMP. cGAMP then binds to STING on the ER to trigger STING trafficking to vesicles. cGAMP-bound STING activates the downstream kinases TBK1 and IKK to activate the transcription factors IRF3 and NF-κB, respectively. These transcription factors induce expression of type I IFNs and cytokines, which propagate the immune response in an autocrine and paracrine manner.

The innate immune system provides the first line of defense against pathogen infection. Pathogen recognition receptors (PRRs) initiate innate immune responses by binding to corresponding pathogen- or damage-associated molecular patterns. cGAS was first discovered as a cytosolic PRR that detects pathogen DNA (Fig. 1). While self-DNA is compartmentalized in the nucleus or mitochondria, pathogen DNA is released into the cytosol during infection of cells. cGAS binds to this DNA in the cytosol and converts ATP and GTP into 2′3′-cyclic GMP-AMP (cGAMP).^6–8^ cGAMP functions as a second messenger that binds to the adapter protein stimulator of interferon genes (STING) on the endoplasmic reticulum (ER) membrane.^7,9–12^ Upon cGAMP binding, STING traffics from the ER to the Golgi apparatus and activates TANK-binding kinase 1 (TBK1) and IκB kinase (IKK).^13^ These kinases activate the transcription factors interferon regulatory factor 3 (IRF3) and NF-κB, respectively, to induce the production of type I interferons (IFNs) and other cytokines.^14,15^ These cytokines orchestrate immune responses to eliminate pathogens such as DNA viruses, retroviruses, and intracellular bacteria.^16–19^ In addition, STING activation induces autophagy to clear intracellular pathogens in a TBK1-independent manner.^20,21^ cGAS and STING are tightly regulated through transcriptional regulation, post-translational modifications, and protein degradation as noted in a previous review.^16^

As cGAS binds the backbone of double-stranded DNA without sequence specificity,^22,23^ cGAS can also be activated by cytosolic self-DNA that leaks out from membranous organelles. Intracellular DNases prevent cGAS from detecting self-DNA by reducing cytosolic DNA levels; three prime repair exonuclease 1 (TREX1) and deoxyribonuclease II (DNase II) degrade DNA in the cytosol and lysosomes, respectively. In mice lacking either of these DNases, cGAS is activated by self-DNA and the mice develop severe autoimmune diseases.^24,25^ Similarly, patients with gain-of-function mutations in STING or loss-of-function mutations in TREX1 or DNase II showed an overactive cGAS-STING pathway and severe autoimmune phenotypes.^26,27^ Additional studies found that cGAS is involved in diseases characterized by “sterile inflammation” such as heart failure, fibrosis, geographic atrophy, and cancer.^28–30^

## The role of cGAS in antitumor immunity

### Cancer immune surveillance

Cancer cells acquire abnormal features such as uncontrolled proliferation by accumulating mutations. Although cancer cells originated from endogenous tissues, the immune system recognizes cancer cells as “foreign cells” and target them for destruction. This concept of “cancer immune surveillance” was first recognized in cases of immunodeficiency in which immunocompromised patients or mice presented with higher risks of developing tumors.^31,32^ Subsequent studies showed that tumor antigen-specific CD8^+^ T cells infiltrate tumor sites where they selectively kill cancer cells.^3–5^ Activation of CD8^+^ T cells requires two signals from antigen-presenting cells: the tumor-specific antigen and co-stimulatory molecules. Tumor-specific antigens derive from cancer cells that express abnormal proteins. While co-stimulatory molecules were known to arise from activation of PRRs and the downstream immune signaling pathway, the cancer-specific pathway was not yet determined.

After type I IFNs were shown to be associated with CD8^+^ T cell activation in cancer patients,^33^ additional studies determined that they stimulated CD8α^+^ dendritic cells to activate CD8^+^ T cells.^34,35^ Several PRRs such as the toll-like receptors (TLRs), RIG-I-like receptors (RLRs), and cGAS can induce type I IFNs upon activation. However, only STING-deficient mice showed defective tumor-specific CD8^+^ T cells and accelerated tumor growth, suggesting that the cGAS-STING pathway is a major pathway that spontaneously detects cancer.^36^

**Fig. 2:**
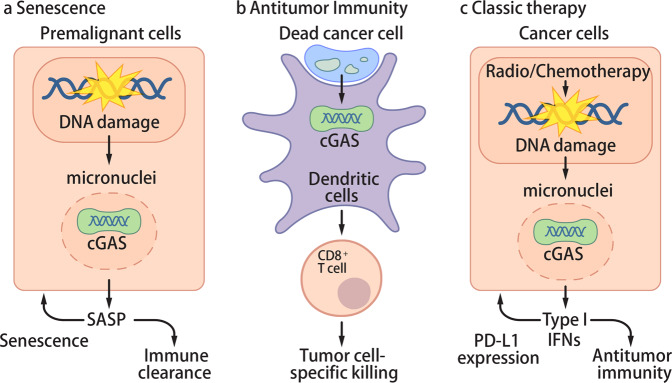
Antitumor roles of cGAS. **a** Premalignant cells acquire DNA damage during tumorigenesis, subsequently forming micronuclei. DNA in micronuclei are exposed to the cytosol and activate cGAS. cGAS induces cytokines and promotes SASP, which enhances senescence and promotes immune cell-mediated clearance of premalignant cells. **b** Tumor-derived DNA from dead cancer cells activate cGAS in dendritic cells. Stimulated dendritic cells prime spontaneous antitumor immunity by activating tumor-specific CD8^+^ T cells and NK cells to kill cancer cells. Similarly, tumor-derived cGAMP from cancer cells is transported to non-cancer cells and activates STING to induce antitumor immunity. **c** Classic cancer therapies (radiotherapy or chemotherapy) induce DNA damage and micronuclei formation. cGAS in cancer cells is activated by micronuclei to induce the production of type I IFNs and other cytokines; although these cytokines enhance antitumor immunity, they also up-regulate PD-L1 expression on cancer cells.

DNA or cGAMP from tumors activates the cGAS-STING pathway to initiate antitumor immunity. Tumor DNA was detected in the cytosol of host cells and subsequently induced type I IFNs in dendritic cells and endothelial cells.^36,37^ The mechanism by which tumor DNA is transferred to the cytosol of non-tumor cells remains to be resolved. Due to genome instability, some tumor cells spontaneously produce cGAMP, which is transferred to non-tumor cells; tumor cGAS and host STING were required for antitumor immune responses, supporting the cGAMP transfer model.^38,39^ So far, gap junctions, SLC19A1, P2X7R, and LRRC8 were reported to transmit cGAMP from cell to cell or from the extracellular region to cells.^40–45^ Altogether, spontaneous cancer immune surveillance is induced by tumor-derived DNA or cGAMP transferred into host cells (Fig. 2b).

Although the immune system has a critical antitumor effect, persistent inflammation can promote tumor growth and metastasis.^46^ In an inflammation-driven epithelial cancer model, the carcinogen DMBA activated the cGAS pathway to induce inflammation that promoted tumorigenesis; STING-deficient mice were resistant to DMBA-induced tumorigenesis.^47^ In a brain tumor model, cGAMP generated in cancer cells was transferred to astrocytes through gap junctions to induce inflammation and metastasis.^48^ Thus, the cGAS-STING pathway also has protumor functions by promoting inflammation-driven tumorigenesis and metastasis. The extent of the protumor effect may depend on levels of genome instability and cGAS activation in tumor cells. Despite the protumor aspect of inflammation, acute activation of immunity was shown to have a strong antitumor effect. As a widely recognized endogenous sensor of tumors, cGAS and its downstream signaling pathway are strong therapeutic targets for cancer immunotherapy.

### Cancer immunotherapy

Cancer immunotherapy focuses on enhancing antitumor immune responses to target cancer cells specifically. In the late 19th century, inactivated bacteria was shown to reduce sarcomas in patients.^49^ Further groundbreaking findings in cancer immunology led to the development of several immunotherapies, including immune checkpoint blockade.^50^ Programmed cell death protein 1 (PD-1) on the T cell membrane interacts with its ligand programmed death-ligand 1 (PD-L1) to prevent over-activation of T cells.^51^ Cancer cells induce PD-L1 expression to suppress tumor-specific T cells, but this immune evasion can be overcome by blocking the interaction between PD-1 and PD-L1. Antibodies targeting PD-1 or PD-L1 to prevent cancer immune tolerance are FDA-approved for treating several tumors.^52^

Despite successful treatment of many cancer patients with immune checkpoint inhibitors, the overall response rate of the therapy is low.^53^ Fostering a CD8^+^ T cell-rich tumor environment may enhance the responsiveness of immune checkpoint inhibitors. As an endogenous pathway for tumor-specific T cell activation, the cGAS-STING pathway is a potent therapeutic target. Importantly, cGAS was essential for the therapeutic effect of PD-L1 antibody as no therapeutic effect was observed in antibody-treated cGAS-deficient mice implanted with tumors: this is due to the need for activation of tumor-specific T cells to precede immune checkpoint inhibitor therapy.^54^

Many therapeutic strategies targeting the cGAS-STING pathway have been developed and tested in preclinical models.^55^ 2′3′-cGAMP, the endogenous ligand of STING, reduced implanted tumor growth by activating and recruiting CD8^+^ T cells to the tumor microenvironment.^37,54^ Combining cGAMP and immune checkpoint inhibitors induced a synergistic effect in controlling tumor growth, demonstrating that activation of STING can potentiate the effect of immune checkpoint inhibitors.^37,54,56^ Although cGAMP has been determined to have a potent antitumor effect, it is degraded by ecto-nucleotide pyrophosphatase/phosphodiesterase (ENPP1) in the serum.^57^ This finding led to the development of STING agonists that are resistant to ENPP1 degradation. In contrast to the results obtained from mouse tumor models, these STING agonists alone did not show prominent antitumor effects in early clinical trials; nevertheless, outcomes were more promising when combined with an immune checkpoint inhibitor (NCT03010176, NCT03172936). As patient tumors have a diverse genetic background and engage multiple immune evasion mechanisms compared to implanted mouse tumors, combining multiple cancer treatments together with STING agonists may be a better approach for patient treatment. In this regard, other STING agonists also enhanced the antitumor effect when combined with tumor vaccines, chemotherapy, or radiotherapy: such applications of STING agonists are expected to continue to expand.^55^ Moreover, the newly identified role of cGAS in the DNA damage response (DDR) widens the scope of immunotherapies (see below).

## The role of cGAS in the DNA damage response

DNA, the blueprint of life, is protected by a series of DDR pathways to maintain genomic integrity. DDR pathways halt the cell cycle and repair DNA; the cell cycle resumes after repairs. If the damage is not resolved, cells undergo an irreversible cell cycle arrest called cellular senescence.^58^ If DNA is left severely damaged, the DDR directs cells to undergo apoptosis. Although the type of DNA damage varies, micronuclei are a traditional biomarker of DNA damage and chromosome instability.^59^ A micronucleus is a small nucleus-like body that is composed of fragments of chromosomes surrounded by a fragile nuclear envelope. They can be generated during mitosis from chromatid fragments formed by DNA double-strand breaks (DSBs) or from lagging chromosomes formed by mis-segregation.^60^ Although micronuclei have been extensively studied since their discovery in the 19th century, their physiological function has remained obscure and micronuclei were simply treated as a readout of genome instability. However, the discovery of cGAS activation in the micronuclei provides a link between DNA damage and innate immune responses.

Multiple studies have now reported that micronuclei activate cGAS.^61–65^ The nuclear envelope of micronuclei easily ruptures due to lack of a stable nuclear lamina.^66^ Micronuclear DNA co-localized with cGAS after micronuclear membrane collapse: this colocalization was enhanced when Lamin B1 was downregulated, indicating that the rupture of the micronuclear membrane precedes DNA detection by cGAS.^63,65^ As premalignant cells accumulate micronuclei due to their unstable genome, subsequent activation of cGAS can promote cellular senescence.

Chromatin in micronuclei is presumably the ligand of cGAS. Indeed, cGAS shows affinity for chromatin and co-localizes with nuclear chromatin during mitosis after nuclear envelope breakdown.^64,67,68^ However, nuclear chromatin, unlike micronuclear chromatin, does not activate cGAS. Micronuclear DNA accumulate DSBs that may reveal cGAS ligands; a structural study of cGAS–DNA complexes suggested preferential binding of cGAS to the terminal regions of dsDNA.^22^ Fragmentation of chromatin may also remove nucleosome packing, which inhibits cGAS activation.^68^

Endogenous defects in the DDR can also activate cGAS and induce autoinflammatory diseases. Aicardi-Goutières syndrome (AGS) is a severe neurodevelopmental disorder characterized by high levels of type I IFNs. AGS patients harbor mutations in genes encoding enzymes involved in nucleic acid metabolism, including TREX1, SAMHD1, and RNaseH2.^69^ SAMHD1 is a dNTPase that restricts reverse transcription of retroviruses and facilitates exonuclease function during DSB repair; SAMHD1 deficiency leads to DNA damage that activates the cGAS pathway.^70^ RNaseH2 excises misincorporated ribonucleotides from DNA; its deficiency induces micronuclei formation and cGAS activation to cause AGS.^63^ Removal of STING or cGAS rescued RNasH2-mutant mice from autoinflammatory phenotypes.^71^ The genetic disease ataxia-telangiectasia (A-T) is caused by dysfunction of the ataxia-telangiectasia mutated (ATM) protein, a central mediator of DSB repair.^72^ As a result, the adaptive immune system, which requires DNA recombination for T and B cell development, is defective in A-T patients.^73^ In contrast, innate immune responses are over-activated and produce excessive amounts of type I IFNs. The cGAS-STING pathway was found to be responsible for this severe inflammation as genetic ablation of STING in the ATM-deficient mouse model greatly reduced the IFN signature.^74^

While micronuclear cGAS detects DNA damage and induces immune responses, nuclear cGAS was recently shown to suppress DNA repair in a STING-independent manner. Homologous recombination, a critical step of DSB repair, was inhibited when nuclear cGAS compacted DNA or interfered with the function of a DNA repair enzyme.^67,75^ As genome instability promotes tumorigenesis, nuclear cGAS may have a protumor function.^67^ As cGAS in tumor cells has both anti- and protumor roles based on its localization, it is important to understand how cGAS localization is regulated among tumor cells.

## The cGAS-STING pathway and cellular senescence

Cellular senescence is a state of irreversible cell cycle arrest that occurs under severe cellular stress. The senescent state is naturally induced after cells undergo multiple rounds of proliferations due to shortening of telomeres or accumulation of DNA damage.^76^ It can also result from exogenous stress such as oxygen radicals and radiation. These senescence signals activate the p53 or p16-retinoblastoma protein pathways to halt the cell cycle.^76^ The senescence state was predominant in premalignant tumors and was essential for suppression of tumorigenesis.^77–79^ In addition to halting their own proliferation, senescent cells secrete inflammatory cytokines, growth factors, and proteases, a phenotype termed the senescence-associated secretory phenotype (SASP). The SASP reinforces senescence growth arrest in an autocrine manner and spreads growth inhibition in a paracrine manner.^80^ In addition, chemokines from the SASP can activate and recruit immune cells to eliminate aberrant cells harboring DNA damage.^81^

Multiple studies reported that DNA damage sensing by the cGAS-STING pathway is critical for the SASP (Fig. 2a).^61,62,64,65^ cGAS- or STING-deficient cells showed reduced senescence after serial passage, irradiation, treatment of DNA-damaging drugs, or oncogene expression; these senescence activators also induced micronuclei, which activate cGAS.^61,62,64,65^ In the oncogene model, RasV12-expressing premalignant hepatocytes induced the SASP and were then eliminated by immune cells.^82^ In the absence of cGAS or STING, the SASP and immune cell infiltration were defective; moreover, impaired clearance of RasV12-expressing cells eventually led to the development of tumors.^61,65^ In the colitis-associated cancer model induced by chronic DNA damage and inflammation, mice lacking STING were more susceptible to tumors.^83,84^ These studies indicate that the cGAS-STING pathway-induced SASP may prevent tumorigenesis by reinforcing senescence or augmenting immune cell-mediated clearance of aberrant cells.

Consistent with the role of the cGAS-STING pathway in senescence and tumorigenesis, several cancer cells and immortalized cells downregulate cGAS or STING expression.^6,85^ Low levels of cGAS or STING in cancer cells are associated with poor prognosis of lung adenocarcinoma and hepatocellular carcinoma, suggesting cell-autonomous tumor-suppressing functions of the cGAS-STING pathway.^64,86^ In addition to the SASP, STING-induced autophagy may provide an additional barrier against tumorigenesis in senescence-bypassed cells by inducing cell death; cGAS- or STING-deficient cells escaped autophagic cell death induced by telomeric DNA damage and continued to proliferate.^87^ Further studies are needed to understand the role of STING-induced autophagy in tumors as autophagy is known to prevent tumorigenesis by removing DNA damage inducers but is also known to support tumor cells by providing cellular building blocks and energy.^88^

Despite the importance of cGAS in preventing tumorigenesis, loss of cGAS expression alone does not induce tumors.^64^ This is consistent with the requirement for mutations in multiple oncogenes and tumor suppressor genes in order for a normal cell to transform into a malignant cancer cell. cGAS may exert tumor suppressive effect by providing additional barriers for premalignant cells that are exposed to chronic DNA damage or have mutations in oncogenes or tumor suppressor genes. Even with these barriers against tumorigenesis, some premalignant cells overcome senescence to form tumor cells. When cGAS is continuously activated in these tumor cells by cytosolic DNA, the canonical and non-canonical NF-κB pathways are induced, leading to chronic inflammation that promotes tumor growth and metastasis.^61,89^ These studies suggest that the relation between cGAS and tumors may depend on tumor type and the stage of tumorigenesis.

## The cGAS-STING pathway and radiotherapy of cancer

Radiotherapy, which uses ionizing radiation to induce DSBs and cell death, is given to ~50% of cancer patients and is a major cancer treatment along with surgery and chemotherapy.^90^ Interestingly, in certain cases, radiotherapy shrank tumors that are not directly irradiated (abscopal effect), indicating that DNA damage-induced cell death is not the sole mechanism of radiotherapy.^91^ Later studies revealed that the immune system, particularly CD8^+^ T cells, plays a role in the therapeutic effect of radiotherapy.^92^ In addition, radiotherapy induced type I IFNs at the tumor site, and type I IFN receptors on immune cells were critical for the effect of radiotherapy.^93^ Subsequent studies show that the cGAS-STING pathway promotes antitumor immunity after radiotherapy in two ways: detection of DNA damage in cancer cells and increased detection of tumor-derived DNA in immune cells (Fig. 2b, c).

**Fig. 3:**
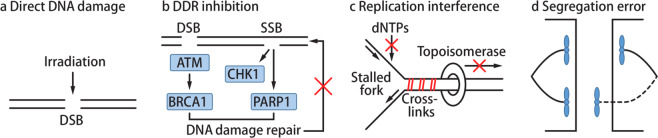
cGAS-activating classic cancer therapies. **a** Irradiation causes DNA breaks and damages. **b** Inhibiting key mediators of the DDR such as PARP1, ATM, and CHK1 leads to accumulation of DNA damage and generation of cytosolic DNA. **c** Interfering with replication halts replication forks, activating DDR and causing DNA breaks. Topoisomerase inhibitors, DNA crosslinkers, and antimetabolites stall replication forks. **d** Anti-microtubule drugs can induce chromosome mis-segregation, leaving a whole or a part of chromosome in the cytosol in the form of micronuclei or cytoplasmic chromatin fragments. All these different cellular perturbations can lead to cGAS activation.

Multiple studies showed that radiation-induced DNA damage causes the formation of micronuclei that then activate the cGAS-STING pathway (Fig. 3).^61–65^ To study whether cGAS activation in irradiated cancer cells contributes to antitumor immunity, the abscopal effect of radiation was investigated. A mouse model was implanted with tumors on one side and injected with irradiated cancer cells at the other side.^62^ Injection of irradiated cancer cells intensified the antitumor immune responses and reduced the contralateral tumor size when combined with an immune checkpoint inhibitor; this effect required STING expression in irradiated cancer cells.^62^ Moreover, direct radiation of implanted STING-deficient tumors did not provide an abscopal effect. This study suggests that cancer cell-intrinsic activation of the cGAS-STING pathway by radiotherapy promotes antitumor immunity.

Another study showed that radiation-induced cell death activates the cGAS-STING pathway in immune cells and potentiates antitumor immunity.^94^ In this study, implanted tumors were directly irradiated in mouse strains deficient in one of several immune signaling pathways; only tumors implanted in STING-deficient mice were resistant to radiotherapy. Dendritic cells were able to detect irradiated cancer cells in a cGAS-dependent manner and induced high levels of type I IFNs in the tumor microenvironment.^94^ This study suggests that the cGAS-STING pathway in dendritic cells detects more tumor-derived DNA after radiotherapy. Increased detection of tumor-derived DNA is probably due to increased cell death after irradiation, as the phagocytic ability of dendritic cells was required for this detection.^94^

The cGAS-STING pathway provides a link between radiotherapy and activation of antitumor immunity. However, high doses of radiation may adversely suppress the immune system; in fact, the most common side effect of radiotherapy is immune suppression. As radiotherapy damages DNA, fast proliferating cells such as cancer cells but also immune cells are affected. Alternatively, radiation-induced activation of cGAS can turn on a negative feedback loop. When radiation doses were above 12–18 Gy, TREX1, an interferon-stimulated gene (ISG), was induced to degrade cytosolic DNA and downregulate the cGAS pathway.^95^ cGAS activation by radiation also upregulates PD-L1, which suppresses antitumor T cells.^96^

Overcoming these hurdles to activate immune responses will be a future direction of radiotherapy. Preclinical studies showed that repeated irradiation at low doses does not induce TREX1 and mediates tumor rejection.^95^ PD-L1 antibody treatment reversed T cell suppression and showed a synergistic therapeutic effect when combined with radiotherapy.^94^ Moreover, combining radiotherapy and cGAMP elicited stronger tumor-specific CD8^+^ T cell responses and showed complete tumor rejection in 70% of mice with implanted tumors.^94^ Revealing an immunomodulatory role of radiotherapy opens up more possibilities of combining it with other immunotherapies. Clinical trials on combining radiotherapy and immune checkpoint blockade are ongoing and will provide new insights in advancing therapeutic approaches.^97^

## The cGAS-STING pathway and chemotherapy

Chemotherapy, which interferes with cell proliferation, is a major treatment given to cancer patients. It was previously assumed that chemotherapy drugs act directly on cancer cells to induce cell death; however, some studies observed the activation of antitumor immunity after chemotherapy.^98,99^ A later study then concluded that increased cell death by chemotherapy releases danger-associated molecules that can activate the immune system.^100^ More recent studies are now suggesting that chemotherapy drugs may also have direct immunostimulatory effects through activating cGAS in cancer cells (Fig. 2c). A growing number of studies are reporting cGAS activation by micronuclei during chemotherapy treatment. Moreover, cancer cell-intrinsic activation of cGAS promoted antitumor immunity, which was critical for the full therapeutic effect of certain chemotherapy drugs.^101,102^ These new findings shift our paradigm of chemotherapy from only being cytotoxic drugs to also having immunostimulatory functions. This realization now raises the need to reinterpret the role of various chemotherapy drugs and develop new applications for chemotherapy and immunotherapy. In this section, we summarize the chemotherapy drugs that are reported to activate the cGAS-STING pathway and their suggested mechanisms of action (Table 1; Fig. 3).

**Table 1:** Chemotherapy drugs activating the cGAS-STING pathway.

Mechanism of action	Drug	FDA approval	Cellular model tested
DDR inhibition			
PARP inhibitors	Olaparib	PPC, breast, ovarian, ovarian epithelial, fallopian tube cancers	SCLC^a^,^102^ TNBC^a^,^101,105^ NSCLC^105,108^
	Veliparib	IUO	TNBC,^101^ osteosarcoma^101^
	Talazoparib	Breast cancer	TNBC,^101,108^ osteosarcoma,^101^ ovarian cancer^a^,^106^ colon cancer^106,108^
	Rucaparib	PPC, ovarian epithelial, fallopian tube cancers	NSCLC,^105^ TNBC^105^
	Niraparib	PPC, ovarian epithelial, fallopian tube cancers	TNBC,^107^ colon cancer^107^
ATM inhibitor	KU60019	IUO	Microglial cells^117^
CHK1 inhibitor	Prexasertib	IUO	SCLC tumors^a ^^102^
Replication interference			
Topoisomerase inhibitors	Teniposide	Acute lymphocytic leukemia	Melanoma,^125^ colon cancer^a^ ^125^
	Etoposide	SCLC, testicular cancer	Melanoma,^64^ lung fibroblast,^61^ MEFs,^64^ BJ cells^64^
	Camptothecin	IUO	MEF^122^
	Acriflavine	IUO	MEF,^122^ bronchial epithelial cells^122^
	Doxorubicin	MM, AIDS-related KS, ovarian cancer	HeLa^124^
	Proflavine with acriflavine	IUO	MEF^123^
DNA crosslinking agents	Cisplatin	Bladder, ovarian, testicular cancers	Melanoma,^146^ breast cancer,^132^ TNBC,^133^ BJ^85^
	Mitomycin C	Pancreatic adenocarcinoma, gastric cancer	Breast cancer^132^
Antimetabolite	Hydroxyurea	CML, HNSCC	TNBC^133^
Segregation error			
Microtubule-targeting drug	Nocodazole	IUO	MEF,^63^ osteosarcoma^63^
	Paclitaxel	NSCLC, AIDS-related KS, breast, ovarian cancers	Breast cancer,^68,141^ HeLa,^68^ BJ^68^

*PPC* primary peritoneal cancer, *SCLC* small cell lung cancer, *TNBC* triple-negative breast cancer, *NSCLC* non-small cell lung cancer, *IUO* investigational use only, *MM* multiple myeloma, *AIDS* acquired immunodeficiency syndrome, *KS* Kaposi sarcoma, *CML* chronic myelogenous leukemia, *HNSCC* head and neck squamous cell carcinoma.

^a^Activation of the cGAS-STING pathway was found in mouse tumors.

### PARP inhibitor

Cancer cells often have defects in the DDR. For example, breast cancer type 1 susceptibility protein (BRCA1) repairs DSBs by homologous recombination and is often mutated in breast and ovarian cancers.^103^ Loss of BRCA function allows premalignant cells to accumulate mutations and potentially transform into cancer cells. In order to maintain minimal genome integrity, these BRCA-deficient cancer cells rely more on the remaining intact DDR pathways, such as single-strand break (SSB) repair. Poly (ADP ribose) polymerase 1 (PARP1) initiates the repair of SSBs that can develop into detrimental DSBs. BRCA-deficient cancer cells cannot repair DSBs accumulated by PARP inhibition and thus undergo apoptosis.^104^ Several PARP inhibitors such as olaparib, rucaparib, niraparib, and talazoparib are FDA-approved to treat multiple cancers including BRCA-deficient breast and ovarian tumors.

Recent studies showed that these PARP inhibitors induced micronuclei formation in cancer cell lines and induced ISGs in a cGAS- and STING-dependent manner.^101,102,105–108^ In mouse implanted tumor models, PARP inhibitors increased infiltration of CD8^+^ T cells to the tumor site. Moreover, the antitumor effect of PARP inhibitors markedly decreased after CD8^+^ T cell depletion, suggesting that an important therapeutic mechanism of PARP inhibitors is to stimulate the immune system.^101,102^ This immunostimulatory effect of PARP inhibitors was not observed when STING-deficient cancer cells were implanted, indicating that PARP inhibitors activate the cGAS-STING pathway in a cancer cell-intrinsic manner to promote antitumor immunity.^101,102^ Activation of cGAS was stronger in BRCA-deficient cancer cell lines, which accumulate more DNA damage upon PARP inhibition.^101,108^ Nevertheless, several studies observed some activation of the cGAS-STING pathway in BRCA-proficient cancer cell lines, possibly due to minor DNA damage.^102,106^ Clinically, PARP inhibitors have shown benefits in both BRCA-proficient and BRCA-deficient tumors.^109,110^ Antitumor immunity induced by cGAS activation might be one explanation for the clinical benefit found in BRCA-proficient tumors; nevertheless, this hypothesis requires additional testing. PARP inhibitors also activated cGAS in cancer cells defective in DNA excision repair protein (ERCC1), which is involved in both nucleotide excision repair and DSB repair.^105^ Altogether, these studies show that PARP inhibitors activate cGAS in cancer cells and promote antitumor immunity.

The newly discovered immunostimulatory function of PARP inhibitors offers new insights into improving cancer patient treatments. Activation of cGAS leads to recruitment of CD8^+^ T cells into tumors but also upregulation of PD-L1 expression on PARP inhibitor-treated cancer cells, thereby sensitizing tumors to PD-L1 immune checkpoint therapy.^102,105,106,111^ Treatment with a PARP inhibitor and a PD-1/PD-L1 antibody showed a synergistic antitumor effect in mouse tumor models^102,105,106,111^; combining niraparib (PARP inhibitor) with pembrolizumab (anti-PD-1) had a promising antitumor activity for patients with breast or ovarian cancer.^112,113^ Combining drugs that target different DDR proteins such as PARP and A-T and Rad3-related protein (ATR) further increased micronuclei formation, indicating stronger cGAS activation.^114^ Synergistic effects may not be observed in all cancer treatments since many cancer cells, including several ovarian cancer cells, lack cGAS or STING expression.^85,115^ Nevertheless, PARP inhibition is also able to elicit antitumor immunity by effectively killing cancer cells and releasing more tumor-derived DNA to stimulate the cGAS-STING pathway in immune cells.^116^ In addition, combination of STING agonists with PARP inhibitors may further promote antitumor immunity when cancer cells maintain the DDR pathway or lack cGAS, widening the scope of PARP inhibitor usage.

### ATM inhibitor

Upon DNA damage, ATM phosphorylates downstream mediators to regulate DDR and the cell cycle. Given the essential role of ATM in DSB repair, two ATM inhibitors (M3541, AZD01156) are under investigation in combination with radiotherapy or chemotherapy (NCT03225105, NCT02588105). ATM deficiency leads to the accumulation of cytoplasmic DNA and induces type I IFNs in a STING-dependent manner^74^; accordingly, the ATM inhibitor KU60019 induced cytoplasmic DNA accumulation and STING-dependent cytokine production in microglial cells.^117^ Another study on pancreatic cancer cells observed TBK1 phosphorylation after KU60019 treatment.^118^ ATM-silenced pancreatic tumors showed increased CD8^+^ T cell infiltration and PD-L1 expression, suggesting that ATM inhibition in tumors can induce antitumor immunity.^118^ ATM silencing in the tumor also sensitized the tumor to PD-L1 antibody or irradiation. However, cGAS and STING were found to be dispensable for TBK1 phosphorylation after KU60019 treatment in this study.^118^ Thus, these early results have not yet resolved the question of whether cGAS is involved in ATM inhibitor-induced immune responses. Future studies are needed to understand how ATM inhibitor-induced DNA damage is detected and whether antitumor immunity is critical for the therapeutic effect of ATM inhibitors.

### Checkpoint kinase inhibitor

Checkpoint kinase 1 (CHK1) monitors DNA damage during DNA replication and regulates the cell cycle; thus, inhibition of CHK1 leads to replication fork stalling and DSBs.^119^ Like other signals that cause DSBs, the CHK1 inhibitor prexasertib induced micronuclei in vitro.^102^ The cGAS-STING pathway was activated by these micronuclei and induced expression of ISGs and PD-L1. In a lung cancer mouse model, CD8^+^ T cells were required for the full antitumor effect of prexasertib.^102^ Moreover, combination of prexasertib with a PD-L1 antibody showed a synergistic antitumor effect. Cancer cells deficient in cGAS or STING were resistant to this combination therapy, suggesting that CHK1 inhibitors enhance antitumor immunity by activating the cancer cell’s intrinsic cGAS-STING pathway.

### Topoisomerase inhibitor

Topoisomerase I and II relieve the torsion of DNA during DNA replication, allowing the replication fork to proceed; inhibition of topoisomerase causes replication fork stalling, inducing DSBs and apoptosis.^120^ Cancer cell death is thought to be the major mechanism of topoisomerase inhibition, but an early study showed that topoisomerase inhibition is linked to IRF3 activation.^121^ Recent studies suggest a role for cGAS and STING in topoisomerase inhibitor-induced immune responses.

Topoisomerase inhibitors such as teniposide, etoposide, camptothecin, doxorubicin, proflavine, and acriflavine induced cytosolic DNA in various cell lines, activating the cGAS-STING pathway.^61,64,122–124^ Teniposide induced infiltration of CD8^+^ T cells to the tumor site and controlled tumor growth in a CD8^+^ T cell-dependent manner.^125^ This effect was markedly impaired when STING expression in tumor cells was silenced, indicating that tumor-intrinsic activation of the cGAS-STING pathway is critical for the therapeutic effect of teniposide.^125^ Additionally, teniposide provided a synergistic effect when combined with PD-L1 antibody in mice implanted with tumor.^125^ Another study reports that the topoisomerase inhibitor topotecan can also activate the cGAS-STING pathway in dendritic cells by inducing release of tumor DNA-containing exosomes from cancer cells.^126^ This antitumor effect of topotecan was abrogated in STING-deficient mice, suggesting that cGAS/STING-mediated immune signaling was essential for the therapeutic effect of topotecan.^126^ Altogether, these reports show that topoisomerase inhibitors can activate the cGAS-STING pathway in cancer cells and/or immune cells to enhance antitumor immune responses.

Several studies suggested alternative mechanisms for topoisomerase inhibition-induced immune responses. An early study reported that doxorubicin activates TLR3 in cancer cells to induce ISGs, which was critical for the therapeutic effect.^127^ A more recent study showed that cGAS is essential for a high level of IFNβ induction in doxorubicin-treated cancer cells while a low level of IFNβ is still induced in a ATM-dependent manner.^124^ Another study also suggested that etoposide induces IFNβ in a cGAS-independent but STING-dependent manner; PARP-1 and ATM detected DNA damage and induce a non-canonical STING signaling complex to enhance NF-κB activation in several human cell lines.^128^ Future investigations on the role of the cGAS-STING pathway and other immune signaling pathways in preclinical tumor models will refine our understanding of antitumor immune responses caused by topoisomerase inhibition.

### DNA crosslinking agent

Crosslinking agents form covalent bonds with nucleophilic substrates, preferably a guanine base of DNA, and generate various DNA adducts or crosslinks.^129^ These types of DNA damage halt replication forks, inducing DSBs. Crosslinking agents have been reported to be immunogenic and rely on CD8^+^ T cells for their therapeutic effect.^130,131^ More recently, crosslinking agents such as cisplatin, mitomycin C, and mafosfamide were shown to induce cytosolic DNA and ISG expression in various cancer cells.^85,132–135^ ISG induction was increased in the absence of TREX1, suggesting cGAS involvement.^132^ In the cytosolic fraction of cisplatin-treated cells, cGAS was bound to histone H3, indicating the interaction of cGAS with chromatin released from the nucleus.^133^ Moreover, cGAS and STING were required for induction of ISGs by crosslinking agents.^85,132,133,135^ DNA crosslinking agents upregulated PD-L1 expression on cancer cells, and combining anti-PD-L1, anti-PD-1, or anti-CTLA-4 with cisplatin gave a synergistic effect in treating several tumor models.^133,134,136^ Altogether, these studies show that cGAS promotes an antitumor effect by detecting DNA damage caused by crosslinking agents. The immunomodulatory function of crosslinking agents provides a scientific rationale to combine them with other immunotherapies.

### Antimetabolite

Antimetabolite cancer therapies interfere with DNA replication; one of their targets is ribonucleotide reductase, which generates building blocks of DNA. Inhibiting ribonucleotide reductase with hydroxyurea halts replication forks and causes DSBs.^137^ Hydroxyurea-induced DNA damage upregulates ISG expression in BRCA1-deficient breast cancer cells in a cGAS/STING-dependent manner.^133^ In addition, hydroxyurea treatment increased PD-L1 expression on cancer cells, suggesting that the combination of antimetabolite drugs and immune checkpoint inhibitors may show a synergistic effect. In this regard, the antimetabolite drug 5-fluorouracil showed a synergistic antitumor effect when combined with cGAMP treatment.^138^ Moreover, combining cGAMP treatment reduced the toxicity of 5-fluorouracil, as shown by reduced intestinal damages, suggesting that combination therapies may have additional advantages over mono-chemotherapy.^138^ Several clinical trials are ongoing to determine the combination effect of pembrolizumab, 5-fluorouracil, and cisplatin (NCT02494583, NCT03189719).

### Microtubule-targeting drug

Blocking mitosis was one of the earliest strategies to interfere with cancer cell proliferation. Several microtubule inhibitors such as paclitaxel (Taxol) interfere with chromosome segregation and induce mitotic arrest followed by apoptosis^139^; however, the in vivo contribution of mitotic arrest in the therapeutic effect remains controversial as inhibitors targeting other mitotic processes were not as effective as microtubule-targeting drugs.^140^ At lower concentrations, paclitaxel causes chromosome mis-segregation, which leads to micronuclei formation.^139^ Recent studies found that micronuclei induced by nocodazole or paclitaxel co-localizes with cGAS to induce the expression of downstream cytokines.^63,141^ Given our recent knowledge regarding cGAS activation by micronuclei, activation of cGAS by microtubule-targeting drugs has the potential to induce antitumor immunity.^30^

In addition, the cGAS-STING pathway may have a direct role in promoting cell death via anti-microtubule drugs. cGAS activation by paclitaxel induced type I IFNs and TNFα in breast cancer cell lines thereby driving other cells to apoptosis in a paracrine manner; mechanistically, these cytokines induced the pro-apoptotic regulator Noxa to promote mitochondrial outer membrane permeabilization (MOMP).^141^ High doses of paclitaxel predominantly induce mitotic arrest rather than the micronuclei formation.^68,142^ In another study, paclitaxel-induced mitotic arrest activated cGAS and induced slow phosphorylation of IRF3 that accelerated MOMP.^68^ Consistently, high levels of cGAS expression in non-small cell lung cancer correlated with prolonged survival for paclitaxel-treated patients.^68^ In the human tumor xenograft model using immunocompromised mice, the antitumor effect of paclitaxel depended on the expression of cGAS or STING in cancer cells.^68,141^ Future studies of antitumor immunity and apoptosis in paclitaxel-treated immunocompetent mouse models will help us design additional therapeutic strategies using microtubule-targeting drugs.

## Future perspectives

It is now clear that classic cancer therapies have immune-modulating functions; furthermore, the antitumor immunity was essential for the therapeutic effect of several classic therapies in preclinical studies. These new findings suggest that classic cancer therapies are not merely cytotoxic treatments. The cGAS-STING pathway mediated the interplay between the cytotoxic effect and immune stimulation by detecting DNA damage-induced micronuclei or cytoplasmic chromatin fragments and promoting antitumor immune responses.

The newly discovered role of classic cancer therapies as immune stimulants provides insights into designing therapeutic strategies. For example, the immune-stimulating ability of chemotherapy drugs in development can be monitored together with their cytotoxicity. Chemotherapy drugs that have a better ability in inducing micronuclei formation may have more clinical benefits by activating cGAS and promoting antitumor immunity. New chemotherapy “cocktails” can also be designed to maximize genome instability and the immunostimulatory effect. Furthermore, classic therapies can be combined with immunotherapies to enhance antitumor immunity. Combining classic therapies with immune checkpoint blockade showed synergistic antitumor effects in multiple preclinical tumor models and clinical trials.^96,102,106,111,136^ STING agonists further enhanced the antitumor immunity when combined with classic therapies.^94,138^ Moreover, several studies suggested that activation of the cGAS-STING pathway has additional benefits in promoting immunostimulatory effects of chemotherapy drugs while reducing toxicity.^68,138,141^ It will be interesting to compare the therapeutic effects of different combination therapies and look into their mechanisms of action.

Studies of the cGAS-STING pathway in tumors have also led to new findings about the pathway. The canonical cGAS-STING pathway induces autophagy and IRF3- and NF-κB-mediated cytokine expression. In addition to the canonical NF-κB pathway, non-canonical NF-κB pathway involving p100 and RelB was activated by cGAS and STING. Canonical NF-κB pathway was required for the therapeutic effect of radiotherapy whereas non-canonical NF-κB pathway was inhibitory.^143^ Moreover, persistent activation of non-canonical NF-κB pathway in cancer cells with highly unstable genomes promoted metastasis due to chronic inflammation.^89^ In addition, new studies found non-canonical cGAS and STING pathways that were independent of each other. Nuclear cGAS interfered with DDR by binding to the DNA repair enzyme PARP1 or compacting DNA independently of STING.^67,75^ The DDR pathway involving PARP-1 and ATM led to formation of a STING signaling complex that includes p53 and the E3 ubiquitin ligase TRAF6 to induce cytokines independently of cGAS; unlike canonical STING activation, this novel complex predominantly activated the NF-κB pathway.^128^ New findings in signaling and regulation of the cGAS-STING pathway will allow us to utilize diverse methods to operate this pathway for cancer therapy. For example, specific inhibitors for the non-canonical NF-κB pathway enhanced the antitumor effect of radiotherapy.^143^

The cGAS-STING pathway itself has numerous antitumor roles: promoting senescence in premalignant cells, inducing spontaneous antitumor immunity, and responding to classic cancer therapies. Consistently, acute activation of the cGAS-STING pathway provides an antitumor effect; however, chronic inflammation by persistent and spontaneous activation of STING may promote tumor growth and metastasis. In this regard, the presence of STING and downstream NF-κB signaling in astrocytes and breast cancer cells increased metastasis of brain and breast tumors, respectively.^48,89^ Moreover, STING-induced inflammation promoted inflammation-driven tumorigenesis.^83^ STING activation also induced a negative feedback loop that downregulated immune responses. Spontaneous activation of STING by tumor implantation induced indoleamine 2,3-dioxygenase, which suppressed antitumor immunity.^144^ STING activation by radiotherapy induced TREX1 and recruited myeloid-derived suppressor cells to suppress immune responses.^95,145^ Such immunosuppression can be overcome by regulating the dose and frequency of radiotherapy or by combining with other immunotherapies. Although acute activation of the cGAS-STING pathway predominantly induces antitumor immune responses, the protumor functions of the pathway should be considered during cancer treatments. Further evaluations of the stage of cancer, clinical dose, frequency, and duration are needed to optimize the antitumor effect of the cGAS-STING pathways while minimizing chronic inflammation and immunosuppression.
